# Pharmacokinetic Changes Induced by Focused Ultrasound in Glioma-Bearing Rats as Measured by Dynamic Contrast-Enhanced MRI

**DOI:** 10.1371/journal.pone.0092910

**Published:** 2014-03-26

**Authors:** Feng-Yi Yang, Chia-En Ko, Sheng-Yao Huang, I-Fang Chung, Gin-Shin Chen

**Affiliations:** 1 Department of Biomedical Imaging and Radiological Sciences, National Yang-Ming University, Taipei, Taiwan; 2 Biophotonics and Molecular Imaging Research Center, National Yang-Ming University, Taipei, Taiwan; 3 Institute of Biomedical Informatics, National Yang-Ming University, Taipei, Taiwan; 4 Division of Medical Engineering Research, National Health Research Institutes, Miaoli County, Taiwan; The Ohio State University Medical Center, United States of America

## Abstract

Focused ultrasound (FUS) combined with microbubbles has been shown to be a noninvasive and targeted drug delivery technique for brain tumor treatment. The purpose of this study was to measure the kinetics of Gadolinium diethylenetriamine pentaacetic acid (Gd-DTPA) in glioma-bearing rats in the presence of FUS-induced blood-brain barrier disruption (BBB-D) by magnetic resonance imaging (MRI). A total of ten glioma-bearing rats (9–12 weeks, 290–340 g) were used in this study. Using dynamic contrast-enhanced (DCE)-MRI, the spatial permeability of FUS-induced BBB-D was evaluated and the kinetic parameters were calculated by a general kinetic model (GKM). The results demonstrate that the mean *K*_trans_ of the sonicated tumor (0.128±0.019 at 20 min and 0.103±0.023 at 24 h after sonication, respectively) was significantly higher than (2.46-fold at 20 min and 1.78-fold at 24 h) that of the contralateral (non-sonicated) tumor (0.052±0.019 at 20 min and 0.058±0.012 at 24 h after sonication, respectively). In addition, the transfer constant *K*_trans_ in the sonicated tumor correlated strongly with tissue EB extravasation (R = 0.95), which suggests that DCE-MRI may reflect drug accumulation in the brain. Histological observations showed no macroscopic damage except for a few small erythrocyte extravasations. The current study demonstrates that DCE-MRI can monitor the dynamics of the FUS-induced BBB-D process and constitutes a useful tool for quantifying BBB permeability in tumors.

## Introduction

The blood-brain barrier (BBB) blocks molecules with a molecular weight higher than 180 Dalton due to its physiological and anatomic properties [1], [2]. Hence, most potent therapeutic agents might be ineffective if they cannot reach tumor cells lying beyond the BBB. Despite the fact that the integrity of the BBB is often reduced somewhat near a brain tumor, therapeutic agents are rarely effective in patients with brain tumors because the selective permeability of the BBB still limits the agent reaching the target [3]. Recently, the permeability of the BBB can be temporarily increased by focused ultrasound (FUS) in the presence of an ultrasound contrast agent (UCA) [4], [5]. This effect is reversible, allowing for a time window during which the chemotherapy must be administered to maximize delivery to the target site of the brain tumor. Previous studies have reported that FUS increases the tumor-to-normal tissue ratio for drugs and increases treatment efficacy in the brain tumor [6]–[8]. Additionally, repeated use of FUS may be effective for achieving planed high-dose chemotherapy for brain diseases with minimal systemic toxicity [9], [10].

Dynamic contrast-enhanced MRI (DCE-MRI) is a noninvasive method for calculating the pharmacokinetic parameters of contrast agent distribution within tissues. DCE-MRI has been widely employed in past research to assess kinetics in tumor-bearing rats [11], [12]. The chemical structure, paramagnetic properties, and pharmacokinetics of Gd-DTPA have been described previously [13]. The kinetic models should be adjustable to the properties of the disease to be optimally implemented. In the general kinetic model (GKM), the transfer constant and rate constant between blood plasma and extracellular extravascular space (EES) are defined by *K*_trans_ and *K*_ep_, respectively [14]. When using this modeling approach, a reliable arterial input function (AIF) must be identified to define the concentration of contrast agent in the plasma. Accordingly, vascular permeability can be analyzed quantitatively by DCE-MRI.

FUS-mediated drug delivery requires more information of the mechanism for delivery and models for the transport of drugs across the BBB. The real mechanisms of BBB-D induced by FUS are still unclear. Previous studies have shown that MR imaging signal-intensity changes can be used to determine BBB-D during sonication [15]–[17]. Furthermore, nuclear imaging can be used to quantify the amount of radiotracer extravasation following FUS-induced BBB-D [18], [19]. Dynamic PET imaging was shown to be capable of evaluating lipid materials deposited as the result of sonication [20]. Another study demonstrated that PET scans provide a useful tool for estimating the pharmacokinetics of drugs after administering BBB-D and the optimal therapeutic window for effective radiotherapy [21].

Recently, several studies have characterized the kinetics of the BBB permeability of normal brains after FUS exposure using DCE-MRI [22]–[24]. However, more information is needed to create an optimum theoretical model for various conditions. Evaluations of brain tumor models are also necessary for investigating whether kinetic models can be translated to situations wherein the vasculature is abnormal. Therefore, in this study, F98 glioma-bearing rats were sonicated and DCE-MRI was used to characterize permeability changes as a function of time after BBB-D. Moreover, the correlation between the transfer constant *K*_trans_ and tissue EB extravasation in the sonicated tumor was calculated for further confirmation.

## Results

**Figure 1:**
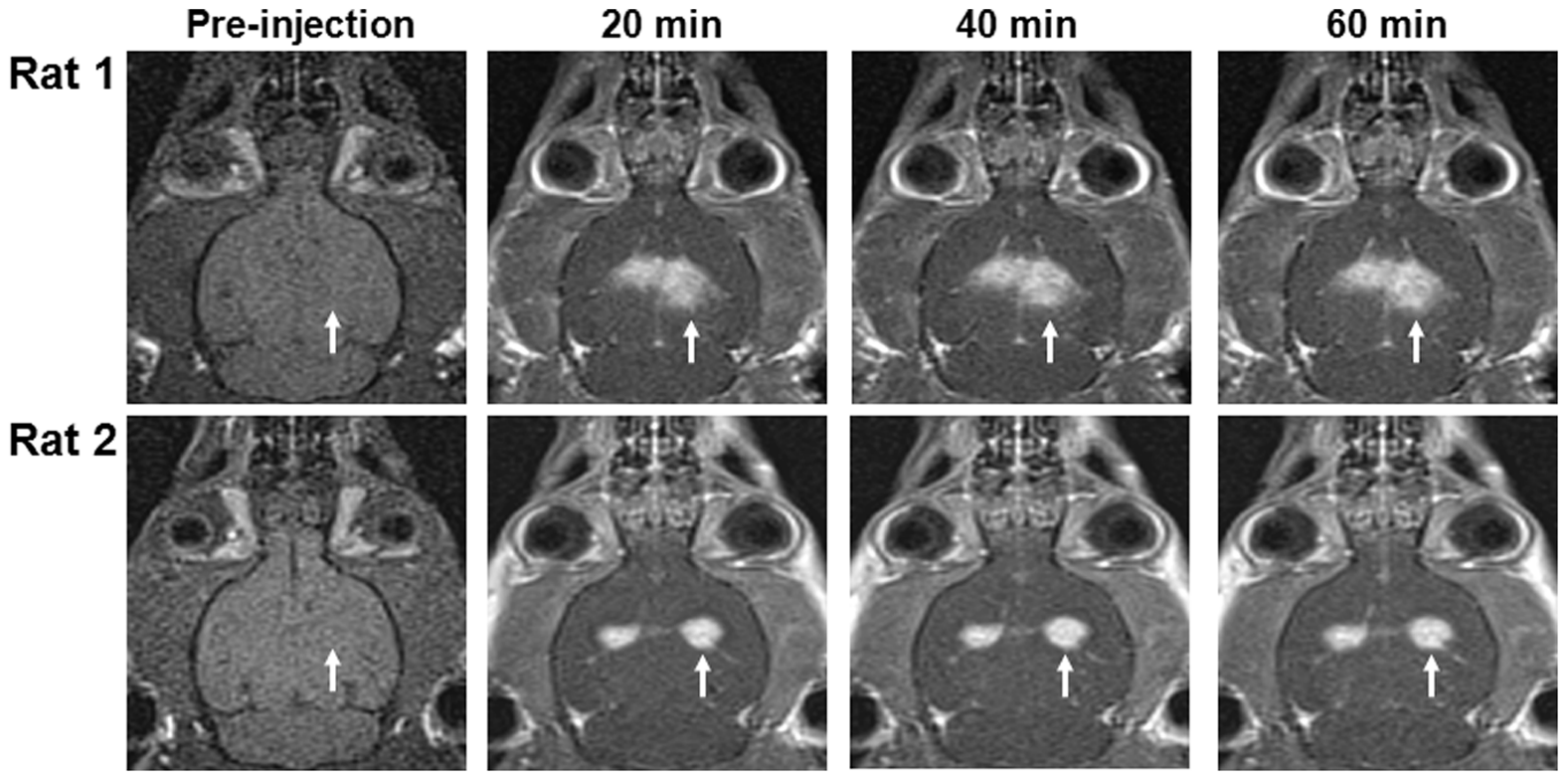
Time series of axial DCE-MRI for FUS-induced BBB-D in brain tumors before and after Gd-DTPA administration. Relative to the control tumor in the left hemispheres, rat 1 and rat 2 show serial contrast changes in the right sonicated brain tumors (arrows) 20 min and 24 h after sonication, respectively.

BBB permeability dynamics in tumors were assessed for a period of approximately 1 h using serial DCE-MRI after Gd-DTPA administration. Figure 1 shows examples of the two glioma-bearing rats 20 min and 24 h after FUS sonication, respectively. Prior to the Gd-DTPA injection, no enhancement was found in the T1-weighted MR images (Fig. 1, left column). Compared to the control tumor, the increased BBB permeability in the right sonicated tumor was evident from a higher signal enhancement in the MR images, especially 20 min after sonication. This finding confirms that BBB permeability decreased over time following FUS-induced BBB-D.

**Figure 2:**
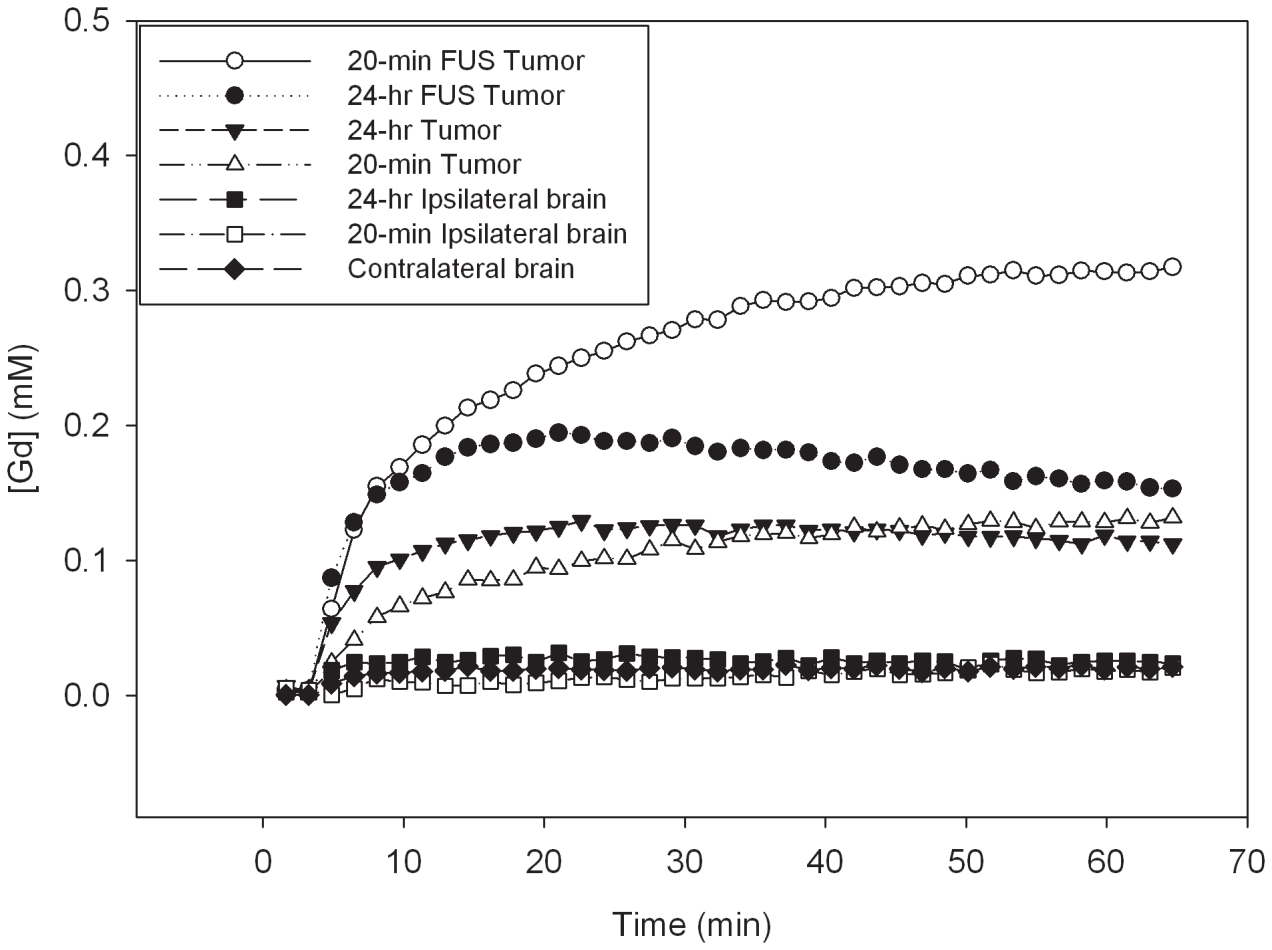
Gd-DTPA concentration as estimated from the ROI in the brain tumor 20 min and 24 h after sonication, ipsilateral brain, and contralateral normal brain.

The middle cerebral artery (MCA) was selected for determining Gd-DTPA concentration in the blood plasma and, subsequently, the parameters of AIF. The AIF was accurately fitted to the bi-exponential equation to yield the mean values of the amplitude constants and decay rates: (*A*_1_, *m*_1_, *A*_2_, *m*_2_) = (0.168, 0.011, 0, −0.521), which were used for the EES concentration fits. Four groups of brain tumors were divided based on FUS sonication and the duration after FUS treatment: 20-min FUS Tumor, 24-h FUS Tumor, 20-min Tumor, and 24-h Tumor. The 20-min FUS Tumor and 24-hr FUS Tumor groups were the tumors sonicated at 20 min and 24 hr, respectively. The groups of 20-min Tumor and 24-h Tumor were the corresponding contralateral control tumors. Figure 2 shows an estimation of the mean changes in Gd-DTPA concentration in the sonicated tumor, contralateral tumor, and the ipsilateral brain. The 20-min FUS Tumor group had the highest concentration of Gd-DTPA in the tumor site. The estimated curves for the changes in Gd-DTPA concentration for the 20-min FUS Tumor group and the 24-hr FUS Tumor group could also be seen to be higher than those of the contralateral tumor for the 20-min Tumor and the 24-h Tumor. The data point groups in Fig. 2 with the lowest Gd-DTPA concentrations are the mean changes in Gd-DTPA concentration in the left contralateral brain and the ipsilateral brain for the 20-min FUS Tumor and the 24-hr FUS Tumor. No significant differences were found among them.

**Figure 3:**
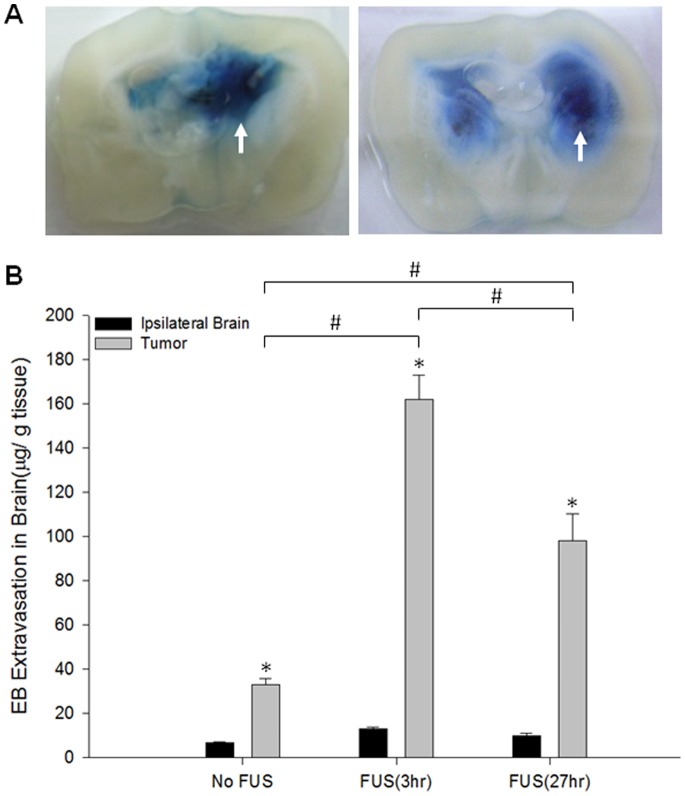
EB extravasation for brain tumors with and without sonication. (A) Distribution of BBB-D in the right brain tumor at 3 h (left column) and at 27 h (right column) after sonication (arrows). Left brain tumor received no sonication as the control tumor. (B) Amount of EB in the control tumor and the sonicated tumor at 3 h and 27 h after FUS exposure. * and # denote significant differences compared with the ipsilateral brain and the tumor, respectively (*^,#^ *P*<0.05; n = 3).

As shown in Fig. 3, EB enters the brain tumors regardless of whether the tumor is sonicated or the control. The representative brain sections indicate that both the size and color intensity of EB in the right sonicated tumors were significantly greater than those in the left control tumors (Fig. 3A). Figure 3B shows the average extravasation of EB (in μg/g of tissue) in the tumor-bearing brains injected intravenously with EB 3 h and 27 h after sonication. In both cases, the degree of EB extravasation in the sonicated tumors was significantly greater than in the unsonicated control tumor. Compared to the tumors injected with EB 3 h after sonication, BBB integrity appeared to have been partially re-established at 27 h, based on the fact that EB administration at this time resulted in a significant difference between the two groups of sonicated tumors.

The mean *K*_trans_, and *K*_ep_ values were derived from DCE-MRIs in the ROI of the sonicated tumor, contralateral tumor, and the ipsilateral brain. Table 1 shows the mean permeability coefficients *K*_trans_ and *K*_ep_ using the GKM as well as *v*_e_ = *K*_trans_/*K*_ep_ and the corresponding correlation coefficient related to EB extravasation. As expected, the mean *K*_trans_ in the 20-min FUS Tumor (0.128±0.019) was significantly higher than in the contralateral tumor (0.052±0.019). However, no significant difference was found for the mean *K*_trans_ between 24-hr FUS Tumor (0.103±0.023) and the contralateral tumor (0.058±0.012) because BBB had been partially recovered at 24 h after FUS exposure. Furthermore, the mean *K*_trans_ in the contralateral 20-min Tumor and the 24-h Tumor were significantly higher than in the respective ipsilateral brains. In addition, there was a higher correlation coefficient (0.95) in the brain tumors between the mean *K*_trans_ and EB extravasation compared with the mean *V*_e_ (0.85).

**Table 1:** Quantitative kinetic parameters estimated from DCE-MRI.

DCE-MRI	Parameters	n	*K*_trans_	*K*_ep_	*V*_e_
FUS	20 min	5	0.128±0.019*	0.032±0.009	4
Tumor	24 hr	5	0.103±0.023	0.092±0.02	1.12
Contralateral	20 min	5	0.052±0.019^#^	0.026±0.013	2
Tumor	24 hr	5	0.058±0.012^##^	0.077±0.016	0.75
Ipsilateral	20 min	5	0.008±0.003	0.049±0.036	0.16
Brain	24 hr	5	0.017±0.004	0.099±0.018	0.17
EB	R		0.95		0.85

R: Correlation coefficient between the parameter and EB extravasation.

* and ^#/##^ denote significant differences compared with the contralateral tumor and ipsilateral brain, respectively (*^,#^ *P*<0.05, ^##^ *P*<0.01).

**Figure 4:**
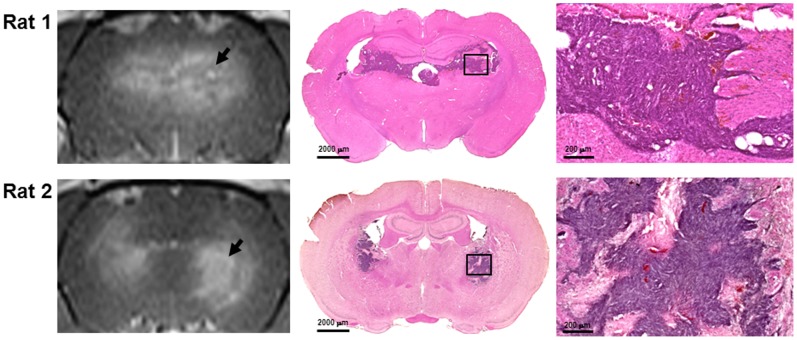
Representative sample of tumors with and without FUS exposure by T1-weighted MR images and hematoxylin-eosin-stained sections on day 8 after implantation. Rat 1 and rat 2 show contrast changes and histology in the right sonicated brain tumors 20&E staining for the whole brain slices. Arrows indicate the brain tumor regions.

MR images were taken to monitor tumor growth noninvasively as a function of time for tumor-bearing rats 20 min and 24 h after sonication (Fig. 4, left column). Histological observation of the brains obtained from tumors 20 min and 24 h after sonication showed mild extravasation of red blood cells in the sonicated tumor tissues in and around the focal region (Fig. 4, middle and right column).

## Discussion

Previous studies have shown that DCE-MRI can be a useful tool for evaluating microvascular permeability and thus drug delivery efficiency through FUS-induced BBB-D in normal brains [22], [24]. This study quantified the permeability of the brain tumor to Gd-DTPA after BBB-D was induced by FUS in the presence of microbubbles. Moreover, EB extravasation was used to assess drug accumulation in the brain tumor after DCE-MRI scanning. These two molecules can be used as a surrogate marker for drug delivery because they normally do not enter the brain tissue from blood capillaries. The results reveal that the transfer constant *K*_trans_ of Gd-DTPA analyzed by DCE-MRI correlated highly with EB extravasation in the brain tumor (*r* ^2^ = 0.95), suggesting that the kinetics of Gd-DTPA by DCE-MRI can potentially estimate the permeability of the BBB and the accumulation of drugs in the brain tumor after FUS exposure.

Leakage of Gd-DTPA through a defective BBB can be measured quantitatively using DCE-MRI [25]. This technology can characterize the permeability of the BBB in the normal brain after FUS exposure. In this study, the transfer constant *K*_trans_ in the brain tumor increased significantly after sonication (Table 1). The ability of sonication to increase BBB permeability for the brain tumor is consistent with the findings of earlier studies, which assessed BBB permeability in the normal brain [24], [26]. However, the rate constant *K*_ep_ showed a mild increase but with no significant differences. It has been proposed that there is a direct relationship between the *V*_e_ = *K*_trans_/*K*_ep_ value and the quantity of materials in the blood vessel that penetrate into the brain tissue due to BBB disruption. The data indicate that FUS exposure mainly increased Gd-DTPA leakage from the systemic circulation to the EES as well as the value of *V*_e_.

Figure 2 shows the Gd-DTPA concentration in the sonicated tumor, contralateral tumor, and the normal brain during a one-hour MRI scan. After Gd-DTPA administration, the concentration in the sonicated tumor and the contralateral tumor showed a rapid increase during approximately the first 15 min and 10 min, respectively. A longer duration was noted for Gd-DTPA delivery into the sonicated tumor than for delivery into the contralateral tumor. While the effectiveness of the BBB may be partially reduced in the area of a brain tumor, Gd-DTPA uptake was nonetheless significantly impeded by the blood-tumor barrier, compared with the sonicated tumor. Quantitative estimation provided numerical permeability values of the BBB, as shown in Table 1. The permeability value *K*_trans_ was higher in the 24-h Tumor than in the 20-min Tumor. This was probably due to the fact that the 24-h Tumor group had an additional day for tumor and vascular growth, relative to the 20-min Tumor group. With the ultrasound parameters used in this study, we did find a few minor erythrocyte extravasation in the sonicated tumor from H&E examination (Fig. 4). Future investigation is necessary for understanding whether any side effects are induced from this minor damage.

Despite the growing number of studies, the mechanism of barrier recovery after FUS-induced BBB-D is still unknown. In particular, the duration of BBB-D has been investigated inadequately. Measuring the therapeutic window during which a drug may entered across the BBB is crucial for estimating the accumulation of drugs released in the target region and to optimize dosage. Recently, one study demonstrated a theroretical model to fit DCE-MRI data and derive a calibration curve to predict the duration of BBB-D in the normal brain [26]. Investigations in brain tumor models are need to confirm whether this methodology used for normal brains can be translated to tumors wherein vasculature growth is abnormal.

In short, this study characterized the kinetics of BBB permeability in the brain tumor model using DCE-MRI and correlated them with measured tissue accumulations of EB in sonicated tumors. The transfer constant *K*_trans_ and the EB accumulations showed a good linear correlation in the brain tumor. To our knowledge, this is the first ever demonstration of the usefulness of DCE-MRI in the quantitative assessment of BBB in brain tumors after sonication. More research is needed to create a pharmacokinetic model for evaluating the permeability of the capillary, thereby optimizing drug delivery through FUS-induced BBB-D.

## Materials and Methods

### Brain Tumor Model

A total of ten Fischer 344 rats (9–12 weeks, 290–340 g) were used in this study. Male Fischer 344 rats (11–13 weeks, 250–280 g) were anesthetized with an intraperitoneal administration of pentobarbital at a dose of 40 mg/kg of body weight. Then, 1×10^5^ F98 rat glioma cells (a generous gift from Dr. Rolf F. Barth, Ohio State University) in 10 μL Hanks’ balanced salt solution without Mg^2+^ and Ca^2+^ were injected into the rats’ brains. The tumor cells were stereotactically injected into a single location in each hemisphere (5.0 mm posterior and 3.0 mm lateral to the bregma) of the rat’s brain at a depth of 5.0 mm from the brain surface. Finally, the holes in the skull were sealed with bone wax, and the wound was flushed with iodinated alcohol. All glioma-bearing rats were treated with sonication in the right brain hemisphere on day 8 after tumor cell implantation. All procedures were performed according to guidelines and approved by the Animal Care and Use Committee of the National Yang-Ming University.

### Focused Ultrasound Setup and Sonication

**Figure 5:**
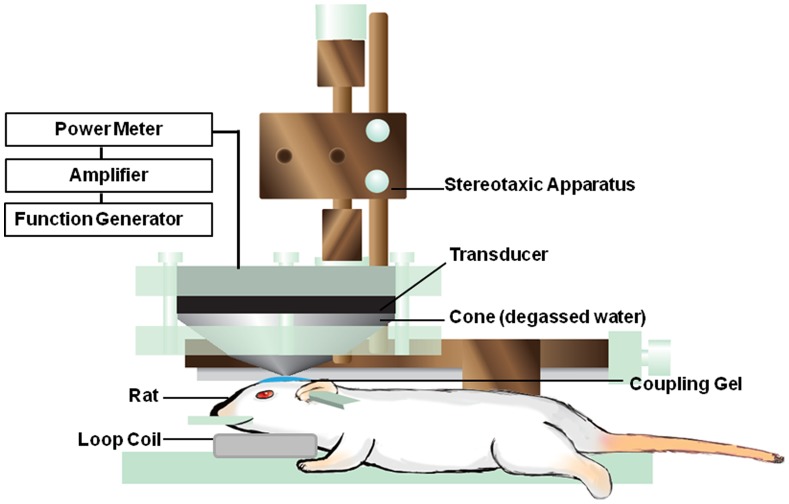
Schematic of the FUS system setup for BBB disruption in rats.

Ultrasound sonication was generated by a 1.04-MHz, single-element focused transducer (H101MR, Sonic Concepts, Bothell, WA, USA) with a diameter of 64 mm and a curvature radius of 62.64 mm. The half-maximum pressure amplitude diameter and length of the focal zone were 1.5 mm and 8 mm, respectively. The transducer was mounted on a removable cone filled with degassed water, and its tip were sealed with a polyurethane membrane. The experimental ultrasound setup was the same as the one used in our previous study [27]. The rats were anesthetized intraperitoneally with urethane (1.2 g/kg) prior to the experiments. UCA (SonoVue, Bracco International, Amsterdam, the Netherlands) was injected into the femoral vein of the rats approximately 15 s before each sonication. The UCA used in this procedure contained phospholipid-coated microbubbles with a mean diameter of 2.5 μm, at a concentration of 1×10^8^ to 5×10^8^ bubbles/ml. The transducer was applied with a burst length of 50 ms at a 5% duty cycle and a repetition frequency of 1 Hz. The duration of the sonication time was 60 s at an acoustic power of 4.1 W and a UCA dose of 150 μL/kg. The ultrasound beam was targeted precisely using a stereotaxic apparatus that used the bregma of the skull as an anatomical landmark. Ultrasound exposure was delivered to one location in the right brain hemisphere, centered on the tumor injection site. The schematic diagram of the FUS system for sonication is shown in Fig. 5.

### MRI Protocol and Signal Analysis

**Figure 6:**
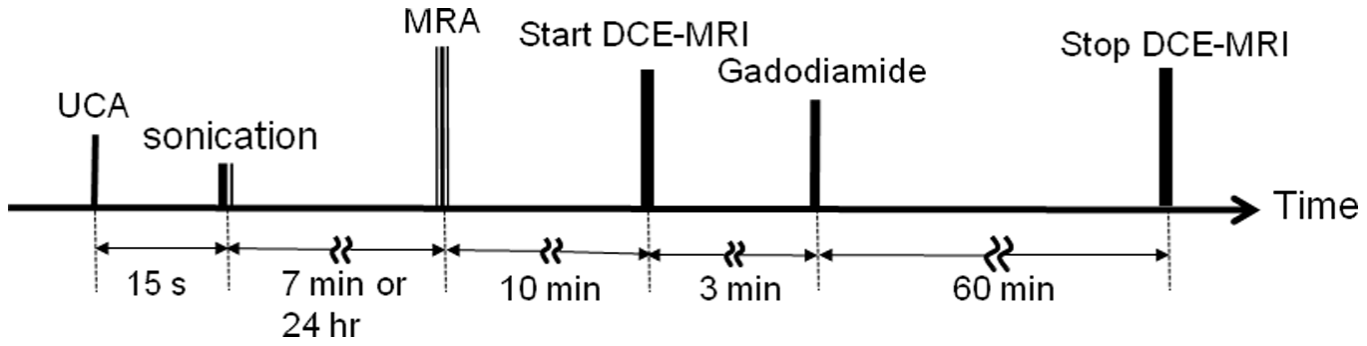
Experimental time line for FUS exposure and the MRI procedure. Fifteen seconds after UCA administration, the sonication was performed before MRA and DCE-MRI scanning.

Each glioma-bearing rat was treated with sonication at the tumor site in the right brain. Immediately or 24 h after sonication, five rats from each group were moved to a 3T MRI system (TRIO 3-T MRI, Siemens MAGNETOM, Germany) for image data acquisition (Fig. 6). A loop coil (Loop Flex Coil, approximately 4 cm in diameter) for RF reception was placed symmetrically under the brain. To detect the position of the MCA for determining the AIF, a set of 3D TOF-MRA (Time-of-Flight magnetic resonance angiography) images was first obtained [28].

Subsequently, 40 sets of T_1_-weighted DCE-MRI images were captured to evaluate the permeability of the BBB in the brain tumor before and after FUS exposure. These 40 sets of DCE-MRI images consisted of the two sets of MRI images taken prior to the Gadolinium diethylenetriamine pentaacetic acid (Gd-DTPA, Omniscan, GE Healthcare, Cork, Ireland) injection (1 mmol/kg), referred to as the initial condition of the brain image, and a total of 38 sets of DCE-MRI images acquired continuously approximately 1 h after Gd-DTPA injection. The parameters of the images for TOF-MRA and T_1_-weighted DCE-MRI are listed in Table 2.

**Table 2:** MRI parameters used in this study.

Sequence	Purpose	FOV(mm)	RT(ms)	ET(ms)	Matrix	FA(°)	SL(mm)	No. of slices	ST
GE	3D TOF-MRA	85×85	14	5.42	256×256	20	0.3	108	10 min 27 s
SE T1-W	DCE	47×80	500	13	152×256	–	1.5	22	64 min 40 s

GE: gradient echo, SE T1-W: spin echo T1-weighted sequence, FOV: field of view, RT: Repetition time, ET: Echo time, FA: Flip angle, SL: Slice thickness, ST: Scan time.

The following procedures were performed for estimating the Gd-DTPA concentration in the sonicated brain region. First, 3 consecutive slices from a set of DCE-MRI images were selected to monitor the sonicated area of the right brain tumor. Then, each of the 3 consecutive DCE-MRI images was set to the region of interest (ROI) of 25 pixels at brighter portions of the sonicated brain tumor. After determining the average strength of the image signals at each of the three ROI, the mean strength of the three averages taken together was calculated to represent the status of the sonicated region in the brain tumor at which point in time the Gd-DTPA penetrated into the tissues due to FUS-induced BBB-D. Finally, the respective mean values from the corresponding DCE-MRI image signals of the 40 points in time were obtained through these two steps, and then a transformation was used to represent the changes in Gd-DTPA concentration in the sonicated brain tumor. Similarly, three ROI of the same size were selected at each point in time in regions of the contralateral brain tumor. The mean value of the signal intensities of the three ROI was calculated for the control tumor group.

### Kinetic Model and Data Analysis

The DCE-MRI images were processed by a GKM, which was a generalized form of the Tofts-Kermode model [25]. Several studies have demonstrated the practicability of the GKM in quantitatively assessing the permeability of FUS-induced BBB-D [22]–[24]. This model is described by the following equation.
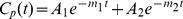
(1)




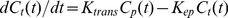
(2)where 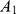 and 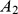 are amplitude constants, *m*_1_ and *m*_2_ are decay rates, and 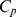 and 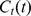 are the Gd-DTPA concentrations at time *t* in the blood plasma and the EES, respectively. The expression of 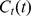 can be obtained by substituting equation (1) into (2):



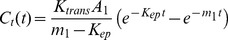
(3)The values of the four constants in Eq. (1) can be estimated by using data regarding the Gd-DTPA concentration of the cerebral artery from 40 points over time. *K*_trans_ and *K*_ep_ values can be calculated from the data regarding Gd-DTPA concentration in the brain tissue region from the same 40 points over time. The Levenberg-Marquardt fitting algorithm in Matlab was used to estimate those variable values for a selected ROI. The Gd-DTPA concentration in tissues can be calculated from the transformation of DCE-MRI signal intensities using the formula:
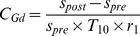
(4)


Here, 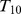 is the T_1_ relaxation time of the arterial blood or the brain tissue before Gd-DTPA administration. The values of 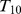 for the brain tissue and arterial blood were 0.9 and 1.5, respectively [29]. 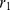 The DCE-MRI relaxivity of the contrast agent 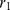 was found to be 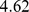 mM^−1^s^−1^ in the 3T MRI system [30]. 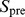
and 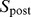 denote the image signal intensities before and after Gd-DTPA injection, respectively [31]. Furthermore, plasma concentration can be calculated as a fraction of 55% blood concentration, 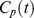 = 0.55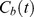.

### Delivery of Evans Blue

BBB permeability can be quantified based on the extravasation of Evans blue (EB), which acts as a marker for albumin extravasation [18], [27]. Here, we used EB to investigate the relationship between the kinetic parameters and the concentration of EB delivered to the sonicated brain tumor. After MRI scanning, EB (Sigma, St. Louis, MO, USA) (100 mg/kg) was injected intravenously 3 h and 27 h following FUS exposure. The rats were sacrificed approximately 4 h after the EB injection. These rats were then perfused with saline through the left ventricle until colorless perfusion fluid appeared from the right atrium. After perfusion and brain removal, the brain was sectioned into three slices (6 mm posterior to the bregma) and mounted on glass slides. The left unsonicated brain tumors acted as the controls. Samples were weighed and then soaked in 50% trichloroacetic acid solution. After homogenization and centrifugation, the extracted dye was diluted with ethanol (1∶3), and the amount of dye present was measured using a spectrophotometer (PowerWave 340, BioTek, USA) at 620 nm. The EB present in the tissue samples was quantified using a linear regression standard curve derived from seven concentrations of the dye; the amount of dye is denoted as the absorbance per gram of tissue.

### Histology

After MRI scanning, two rats from each group were prepared for histological examination. The rats were perfused with saline and 10% neutral buffered formalin. The brains were removed, embedded in paraffin, and then serially sectioned into 30 μm-thick slices. The slices were stained with hematoxylin and eosin (H&E, Thermo-Scientific, Waltham, MA, USA) to visualize their general cellular structure. Photomicrographs of 5 μm-thickness of the H&E staining tissues were obtained using a Mirax Scan digital microscope slide scanner (Carl Zeiss, Mirax 3D Histech) with a Plan-Apochromatic 20/0.8 objective lens.

### Statistical Analysis

The relationship between kinetic parameters (*K*_trans_ and *V*_e_) and EB extravasation was analyzed by calculating correlation coefficients. All values are shown as means ± SEM. Data analysis was performed using an unpaired Student *t* test. Values of *p* ≤ 0.05 were regarded as statistically significant.
