# Correction: Pharmacokinetic Changes Induced by Focused Ultrasound in Glioma-Bearing Rats as Measured by Dynamic Contrast-Enhanced MRI

**DOI:** 10.1371/journal.pone.0106782

**Published:** 2014-08-21

**Authors:** The PLOS ONE Staff

The funding statement for this article is incorrect. Please refer to the correct funding statement below:

This study was supported by grants from the Ministry of Science and Technology of Taiwan (no. NSC 102-2221-E-010-005-MY3 and NSC 101-2320-B-010-036-MY3), Cheng Hsin General Hospital Foundation (no. 103F003C17 and 102F218C11), Biophotonics & Molecular Imaging Research Center, and the Ministry of Education, Aim for the Top University Plan. The funders had no role in study design, data collection and analysis, decision to publish, or preparation of the manuscript.
